# Comparison of standardized uptake value of 18F-FDG-PET-CT with 21-gene recurrence score in estrogen receptor-positive, HER2-negative breast cancer

**DOI:** 10.1371/journal.pone.0175048

**Published:** 2017-04-18

**Authors:** Sung Gwe Ahn, Jae-Hoon Lee, Hak Woo Lee, Tae Joo Jeon, Young Hoon Ryu, Kun Min Kim, Joohyuk Sohn, Mijin Yun, Seung Ah Lee, Joon Jeong, Seung Il Kim

**Affiliations:** 1 Department of Surgery, Gangnam Severance Hospital, Yonsei University College of Medicine, Seoul, Republic of Korea; 2 Department of Nuclear Medicine, Gangnam Severance Hospital, Yonsei University College of Medicine, Seoul, Republic of Korea; 3 Division of Medical Oncology, Department of Internal Medicine, Severance Hospital, Yonsei University College of Medicine, Seoul, Republic of Korea; 4 Department of Nuclear Medicine, Severance Hospital, Yonsei University College of Medicine, Seoul, Republic of Korea; 5 Department of Surgery, CHA Bundang Medical Center, CHA University, Seongnam, Republic of Korea; 6 Department of Surgery, Severance Hospital, Yonsei University College of Medicine, Seoul, Republic of Korea; University of Chicago, UNITED STATES

## Abstract

**Background:**

We investigated the relationship between 18F-fluorodeoxyglucose positron emission tomography-computed tomography (18F-FDG-PET-CT) standardized uptake value (SUV) and 21-gene recurrence score (RS) in estrogen receptor (ER)-positive/HER2-negative breast cancer.

**Materials and methods:**

One hundred sixty-seven patients were identified among those who underwent preoperative 18F-FDG-PET-CT and had RS. Maximum SUV was obtained from 18F-FDG-PET-CT; the cut-off point was 4.

**Results:**

The continuous RS and SUV correlated positively (Pearson’s R = 0.555; *P* < 0.001). An inverse correlation was found between progesterone receptor (PR) expression by reverse transcriptase-polymerase chain reaction, and SUV (Pearson’s R = -0.408; *P* < 0.001). Good agreement between dichotomized RS (<26 vs. ≥26) and SUV (<4 vs. ≥4) was observed in 137 of 167 patients (82.0%; 95% confidence interval [CI], 76.2–87.9). Among patients with low SUV, 114 of 115 (99.1% [95% CI, 97.4–100.0]) had tumors with lower RS (<26). Although 23 of 52 women (44.2% [95% CI, 30.7–57.7]) with high SUV had higher RS (≥26), all 13 women with high RS (≥31) had high-SUV tumors. Most cases with disagreements between SUV and RS (n = 30) were classified as high SUV/lower RS (n = 29). The discordant group had higher grade or elevated Ki67 expression (≥20%) compared with the low SUV/lower RS group (n = 109), but higher PR expression compared with the high SUV/higher RS group (n = 23). Multiple logistic regression analysis showed that high SUV were associated with higher RS (≥26).

**Conclusions:**

SUV, as a biologic parameter represented using a continuous variable, was found to associate with RS in ER-positive, HER2-negative breast cancer. Further studies may reveal the biology underlying the discordance between the markers.

## Introduction

The 21-gene recurrence score (RS), which quantifies the likelihood of distant recurrence in tamoxifen-treated patients with estrogen receptor (ER)-positive breast cancer, was initially developed as a prognostic marker [1]. Subsequently, the RS became the first clinically validated multi-gene assay that could identify patients with ER-positive breast cancer who might benefit from adjuvant chemotherapy [2]. RS has been incorporated into clinical guidelines concerning treatment decisions and has become widespread in actual practice [3,4]. This test has led to sparing patients from chemotherapy and has increased confidence in decision making for patients with early ER-positive cancer [5].

Among patients with hormone receptor (HR)-positive breast cancer, we previously showed that tumors with elevated glucose uptake levels have a higher risk of recurrence [6,7]. In those studies, the prognostic influence of the standardized uptake value (SUV) on 18F-fluorodeoxyglucose positron emission tomography/computed tomography (18F-FDG-PET-CT), which represents glucose uptake, was found to be more significant than subtyping based on immunohistochemical (IHC) markers or tumor burden. Moreover, the associations of high SUV with high histologic grade and high Ki67 index were highly reproducible in luminal cancers [8–10]. Therefore, we postulated that the SUV might positively correlate with the RS.

To address this hypothesis, we investigated the association between the SUV on 18F-FDG-PET-CT as a biologic parameter and RS in patients with ER-positive, HER2-negative breast cancer.

## Materials and methods

### Patients

**Fig 1:**
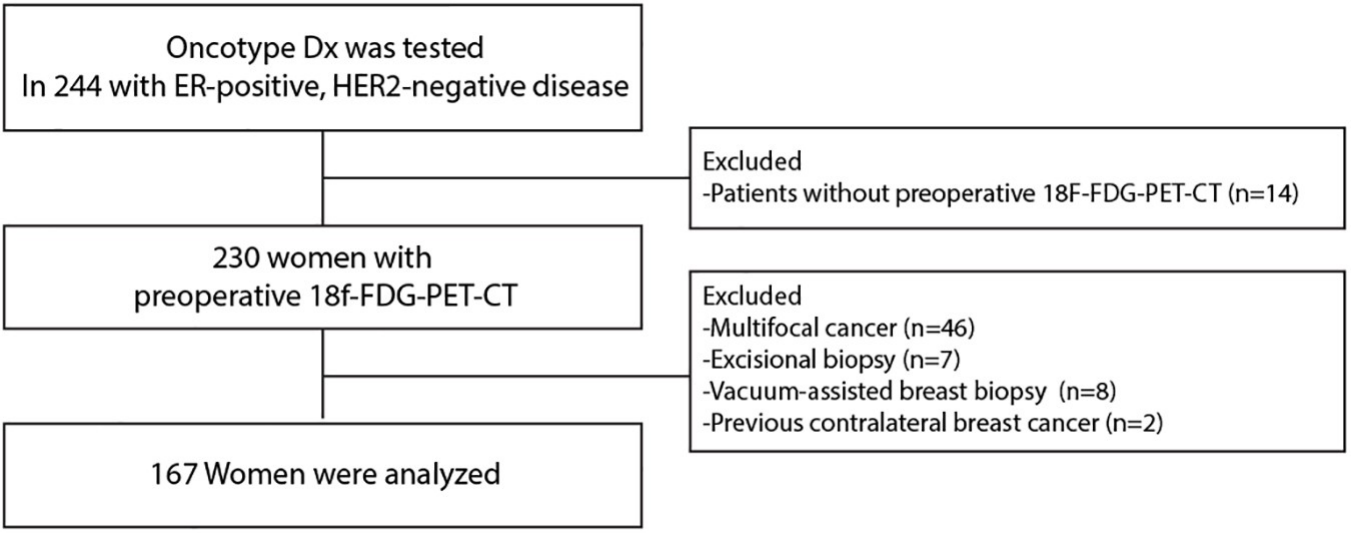
Consort chart.

The institutional review board of Gangnam Severance Hospital, Yonsei University, Seoul, Korea, approved the study to be in accordance with good clinical practice guidelines and the Declaration of Helsinki (3-2013-0146). The need for informed consent was waived due to the retrospective design under the approval of the institutional review board. Between August 2011 and June 2016, 244 patients underwent Oncotype DX testing at Gangnam Severance Hospital and Severance Hospital, Yonsei University College of Medicine. All patients had ER-positive, HER2-negative breast cancer. Of these patients, 230 underwent preoperative 18F-FDG-PET-CT in the context of routine preoperative staging. We excluded multifocal cancers because the resulting tumor heterogeneity might complicate RS testing or SUV determination (n = 46). We also excluded patients undergoing excisional biopsy (n = 7) or vacuum-assisted breast biopsy (n = 8) because these procedures tended to remove large volumes of tumor prior to 18F-FDG-PET-CT imaging. Two patients were excluded because they had a previous history of contralateral breast cancer. Finally, 162 women remained eligible for the analysis (Fig 1).

For our IHC study of four markers, formalin-fixed, paraffin-embedded tissue sections obtained from surgical specimens were stained with appropriate antibodies specific for the ER (1:100 clone 6F11; Novocastra, Newcastle upon Tyne, UK), progesterone receptor (PR; clone 16; Novocastra), HER2 (4B5 rabbit monoclonal antibody; Ventana Medical Systems, Tucson, AZ, USA), and Ki-67 (MIB-1; Dako, Glostrup, Denmark). ER and PR IHC test results were stratified into four groups using the modified Allred system: strong, Allred score 7–8; moderate, Allred score 5–6; weak, Allred score 2–4; and negative, Allred score 0–1 [11]. The HER2 status was considered positive with a score of 3+ and negative with a score of 0 or 1+ [12]. Tumors with a score of 2+ were sent for fluorescent in situ hybridization (FISH) analysis according to the protocol given by the supplier (PathVysion kit; Vysis, Downers Grove, IL, USA or HER2 inform; Ventana). Ki67 expression was measured by an experienced pathologist and presented as a percentage score (range 0–100%) of positive tumor cells.

### Oncotype DX

RS is calculated by the Oncotype Dx assay. It is a continuous score that is classified into the following categories: low risk (RS < 18), intermediate risk (RS 18–30), and high risk (RS≥31). The Oncotype DX assay was performed using RNA extracted from formalin-fixed paraffin-embedded tissue and supplied by Genomic Health (Redwood City, CA, USA). After a review of hematoxylin and eosin-stained slides to determine whether sufficient invasive breast cancer was present and whether manual microdissection was indicated, RNA was extracted from the unstained sections. Cases with no cancer (depleted by prior tissue studies) or with cancer cells occupying <5% of the section area were excluded from the assay [1]. All tissues from patients in this study were successfully analyzed. Quantitative single gene scores for ER and PR mRNA expression, determined via reverse transcriptase-polymerase chain reaction, were also provided by Genomic Health within the final assay report. Normalized ER and PR expression cycle threshold levels (CT) were provided as ER scores and PR scores.

### 18F-FDG-PET-CT

Prior to FDG-PET-CT, each patient was asked to fast for a minimum of 8 hours, and blood glucose levels were controlled to <130 mg/dl. Patients received an intravenous injection of 18F-FDG (5.5 MBq/kg of body weight) in the arm contralateral to the primary tumor. After initial low-dose CT (Discovery Ste, 30 mA, 130 kVp; Biograph TruePoint, 36 mA, 120 kVp), a PET scan was obtained from the neck to the proximal thighs, using a Philips Allegro PET camera (Philips Medical Systems, Cleveland, Ohio, USA) with an acquisition time of 3 min per bed position in three-dimensional mode. The delay between 18F-FDG injection and PET imaging is consistently controlled as 60 minutes. PET images were reconstructed using ordered subset expectation maximization with attenuation correction. For semi-quantitative evaluations, maximum SUV were calculated by measuring the 18F-FDG absorption by tumors in the region of interest. The cross-calibration between the PET and the dose calibrator was conducted monthly. All 18F-FDG-PET-CT scans were reviewed by three nuclear medicine radiologists who were blinded to the RS results.

### Statistical analysis

The primary objective of this study was to test the correlation between continuous RS and continuous SUV. The SUV cut-off point of 4 was determined according to previous studies [6,7]. Pearson’s R was calculated to measure the correlative value between the scores. Discrete variables were compared using the χ2 test or Fisher’s exact test. Student’s *t*-test or a one-way analysis of variation (ANOVA) test was used to compare means. Variables with a statistical significance in the univariate analysis were included in the multiple logistic regression analysis and backward elimination was taken to arrive at the final model. SPSS version 18 (SPSS Inc., Chicago, IL, USA) was used to perform the statistical analyses. Statistical significance was defined as a *P*-value <0.05.

## Results

### Baseline characteristics

The baseline patient characteristics are presented in S1 Table. One hundred and sixty-seven patients with ER-positive, HER2-negative tumors were included in the analyses. The median age of these patients was 48 years (range: 28–72 years). Sixteen patients had node-positive disease, and five had micrometastases. No patient in the study population had a stage higher than IIB.

Ninety-seven (56.8%), 57 (35.2%), and 13 patients (8.0%) had a low, intermediate, and high RS, respectively. Furthermore, 52 patients (32.1%) had tumors with high-SUV (≥4), whereas 110 (67.9%) had tumors with low SUV (<4).

### Correlation between continuous SUV and continuous RS

**Fig 2:**
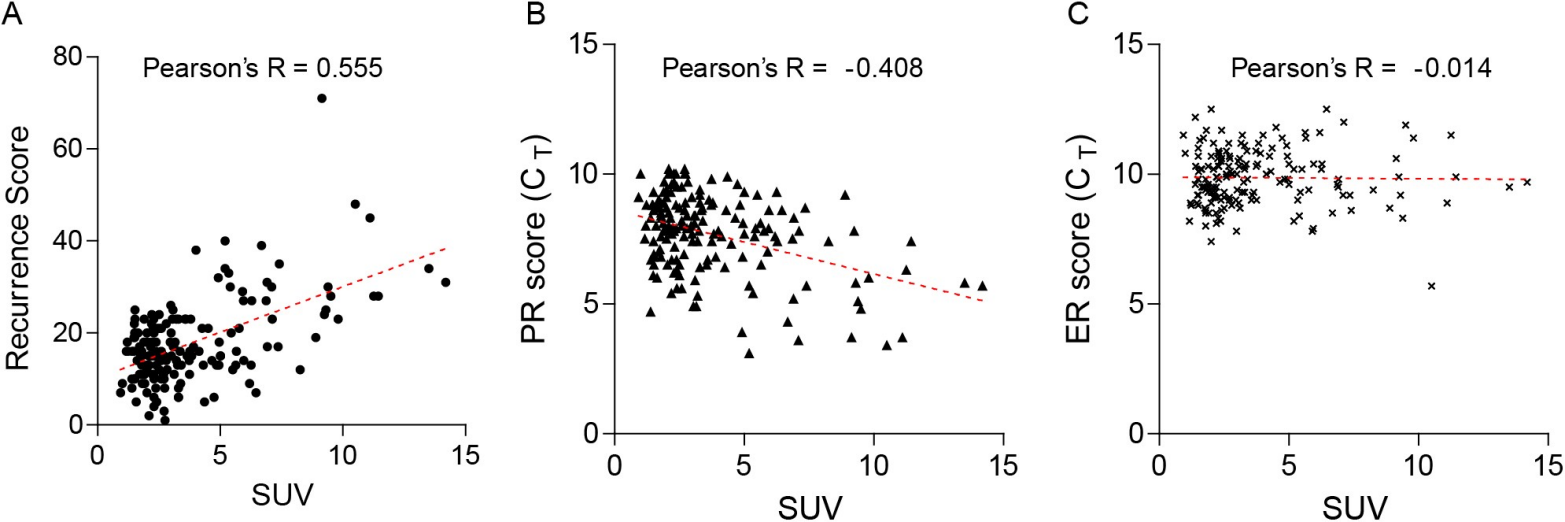
Scatter plots of continuous standardized uptake values (SUV) and continuous recurrence scores (RS). (A) Continuous SUV and continuous RS (Pearson’s R = 0.555; *P* < 0.001); (B) Continuous SUV and continuous progesterone receptor expression (reverse transcriptase-polymerase chain reaction [RT-PCR]) (Pearson’s R = -0.408; *P* < 0.001); (C) Continuous SUV and continuous estrogen expression by RT-PCR (Pearson’s R = -0.014; *P* = 0.856). Footnote: CT, normalized expression cycle threshold levels.

Pearson's R test was performed to explore the relationship between continuous SUV and continuous RS. A significant positive correlation was observed between the two continuous parameters (Pearson’s R = 0.555; *P* < 0.001; Fig 2A). In analyses of ER and PR scores, a certain degree of inverse correlation was observed between the continuous SUV and PR score (Pearson’s R = -0.408; *P* < 0.001; Fig 2B), whereas no significant correlation was observed between the continuous SUV and ER score (Pearson’s R = -0.014; *P* = 0.856; Fig 2C).

### Correlation between categorized SUV and categorized RS

Of the 115 patients with low-SUV tumors, 78 (67.8%) had tumors with a low RS and 37 (32.2%) had an intermediate RS (Table 1). None of the patients had high-RS tumor. Of the 52 patients with high-SUV tumors, 19 (36.4%) had a low RS, 20 (38.5%) had an intermediate RS, and 13 (25.0%) had high RS. All patients with high RS tumors also had high-SUV tumors.

**Table 1:** Concordance between categorized SUV and categorized RS.

		Low SUV (*N* = 115)	High SUV (*N* = 52)
**RS**	Low (*N* = 97)	73 (66.4)	19 (36.5)
	Intermediate (*N* = 57)	37 (33.6)	20 (38.5)
	High (*N* = 13)	0 (0)	13 (25.0)
**d-RS**	Lower-RS (≤25) (*N* = 143)	109 (99.1)	29 (55.8)
	Higher-RS (>25) (*N* = 24)	1 (0.9)	23 (44.2)

Abbreviations: d-RS, dichotomized-recurrence score; SUV, standardized uptake value

Next, RS was dichotomized using a cut-off point of 26, as ongoing clinical trials that have incorporated RS defined high-risk patients as having an RS ≥26 [13]. Of the 115 patients with low-SUV tumors, 114 [99.1% (95% confidence interval [CI], 97.4–100.0)] had tumors with a lower RS. Of the 52 patients with high-SUV tumors, 23 [44.2% (95% CI, 30.7–57.7)] had tumors with higher RS.

**Fig 3:**
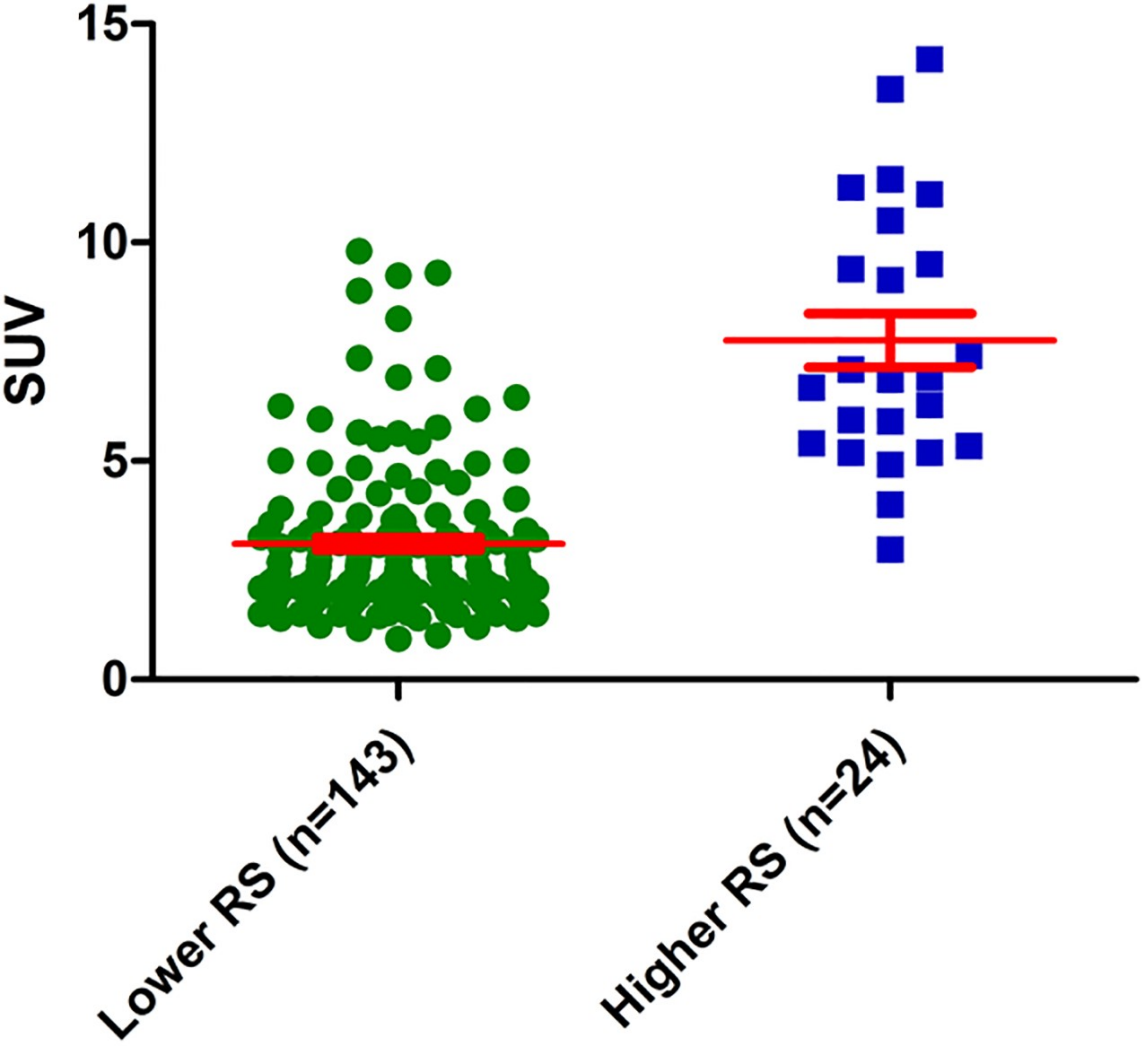
Distributions and means of recurrence scores (RS) according to categorized standardized uptake values (SUV). The mean RS were 14.8 in the low SUV group and 24.0 in the high SUV group (*P* < 0.001).

Moreover, when we compared the mean RS according to dichotomized SUV, a significant difference was observed, with 14.8±5.6 in the low SUV group vs. 24.0±12.1 in the high SUV group (*P* < 0.001; Fig 3).

To identify biological differences between the two markers, we compared characteristics among the groups according to SUV and RS agreement or discordance (Table 2). One hundred and sixty-seven patients were accordingly divided into three groups: low SUV-lower RS (*l*SUV/*l*RS; *n* = 114), discordant SUV-lower RS (discordant; *h*SUV/*l*RS; *n* = 30), and high SUV-higher RS (*h*SUV/*h*RS; *n* = 23).

**Table 2:** Characteristics of the groups, divided by dichotomized SUV and dichotomized RS (n = 167).

		Low SUV-Lower RS (n = 114)	Discordant group (n = 30)	High SUV-Higher RS (n = 23)	*P* ^b^	*P* ^c^	*P* ^d^	*P* ^e^
**Histology**					0.024	0.032	1.000	0.084
	IDC	86 (75.4)	29 (96.7)	22 (95.7)				
	ILC	15 (13.2)	0 (0)	0 (0)				
	Others	13 (11.4)	1 (3.3)	1 (4.3)				
**Tumor size**					<0.001	0.001	1.000	0.001
	≤2cm	101 (88.6)	18 (60.0)	13 (56.5)				
	>2cm	13 (11.4)	12 (40.0)	8 (43.5)				
**Nodal status**					0.710	0.841	0.349	0.452
	Negative	99 (86.8)	25 (83.3)	22 (95.7)				
	Micrometastasis	4 (3.5)	1 (3.3)	0 (0)				
	Positive	11 (9.6)	4 (13.3)	1 (4.3)				
**Stage**								
	IA	87 (76.3)	17 (56.7)	12 (52.2)	0.001	0.006	0.138	0.029
	IB	4 (3.4)	1 (3.3)	0 (0)				
	IIA	22 (19.3)	8 (26.7)	11 (47.8)				
	IIB	1 (0.9)	4 (13.3)	0 (0)				
**Histologic grade**					<0.001	<0.001	0.246	<0.001
	I	36 (31.6)	1 (3.3)	2 (8.7)				
	II	73 (64.0)	21 (70.0)	11 (47.8)				
	III	3 (2.6)	8 (26.7)	10 (43.5)				
	Unknown	2 (1.8)	0 (0)	0 (0)				
**Estrogen receptor** ^**a**^					0.859	0.614	0.488	0.905
	Strong	101 (88.6)	28 (93.3)	20 (87.0)				
	Moderate	10 (8.8)	2 (6.7)	2 (8.7)				
	Weak	3 (2.6)	0 (0)	1 (4.3)				
	Negative	0 (0)	0 (0)	0 (0)				
**Progesterone receptor** ^**a**^					0.002	0.426	<0.001	0.002
	Strong	59 (51.8)	18 (60.0)	3 (13.0)				
	Moderate	28 (24.6)	9 (30.0)	6 (26.1)				
	Weak	12 (10.5)	1 (3.3)	6 (26.1)				
	Negative	15 (13.2)	2 (6.7)	8 (34.8)				
**Ki67**					<0.001	0.006	0.087	<0.001
	<20%	104 (91.2)	22 (73.3)	11 (47.8)				
	≥20%	8 (7.0)	8 (26.7)	12 (52.2)				
	Unknown	2 (1.8)	0 (0)	0 (0)				

^a^ Strong, Allred score 7–8; Moderate, Allred score 5–6; Weak, Allred score 2–4.

^b^ Characteristics among the three groups were compared using the χ^2^ test.

^c^ Characteristics were compared between two groups using the χ^2^ test (Low SUV-Lower RS vs. Discordant SUV-RS).

^d^ Characteristics were compared between two groups using the χ^2^ test (Discordant RS vs. High SUV-Higher RS).

^e^ Characteristics were compared between two groups using the χ^2^ test (Low SUV-Lower RS vs. High SUV-Higher RS).

Abbreviations: SUV, standardized uptake value; RS, recurrence score; IDC, invasive ductal carcinoma; ILC, invasive lobular carcinoma

First, when compared with the *h*SUV/*h*RS group, the *l*SUV/*l*RS group tended to have tumors with a smaller size, lower stage, lower grade, higher PR expression, and lower Ki67 expression, compared with the *h*SUV/*h*RS group.

The discordant group had significantly higher grades and larger tumors, compared with the *l*SUV/*l*RS group (*P* < 0.001 and *P* = 0.001, respectively). In addition, this group had a significantly higher rate of high-Ki67 (≥20%) tumors than did the *l*SUV/*l*RS group (*P* = 0.006). In contrast, PR expression did not differ significantly between the two groups. By contrast, when the discordant group and *h*SUV/*h*RS group were compared, the latter had lower rates of tumors with high PR expression (*P* < 0.001). The groups did not differ with respect to other pathologic factors.

**Fig 4:**
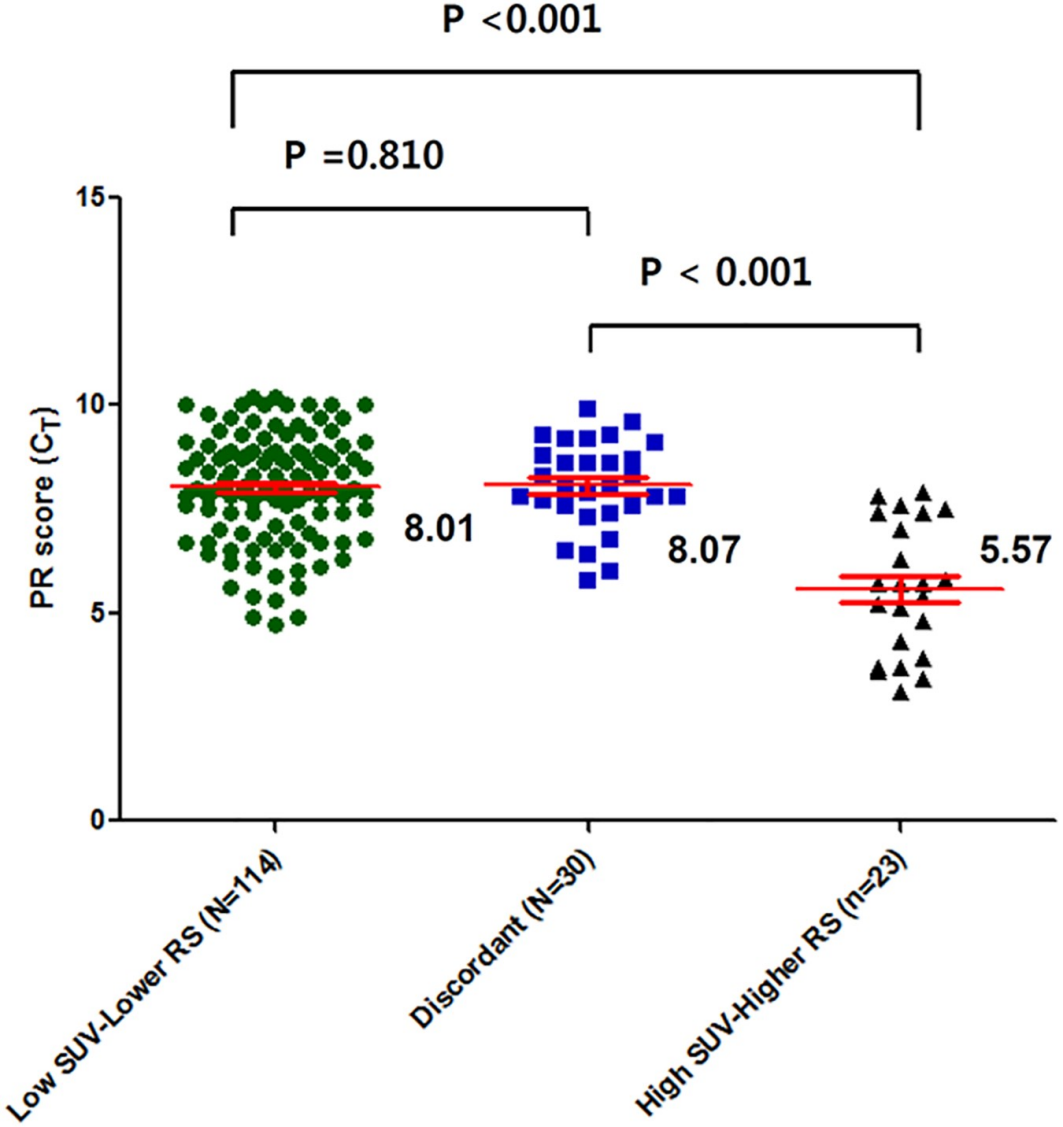
Average PR expression among the three groups, divided by dichotomized SUV and dichotomized RS groups (low SUV-lower RS, the discordant, and high SUV-higher RS). The mean PR expression, determined using reverse transcriptase-polymerase chain reaction, of the high SUV-higher RS was significantly higher than that of the discordant group or the low SUV-lower RS group (*P* < 0.001 and *P* < 0.001, respectively; All *P*-values by the Student’s *t*-test). However, the mean PR scores (C_T_) did not differ between the low SUV-lower RS and the discordant groups (*P* = 0.810). Footnote: CT, normalized expression cycle threshold levels.

Lastly, we compared the mean PR scores among the three groups (Fig 4). This comparison demonstrated the mean PR scores did not differ between the *l*SUV/*l*RS and discordant groups. Collectively, these results indicate that the SUV positively reflects tumor proliferation, as determined via Ki67 or histologic analysis, to a greater extent compared to RS (S1 Fig), whereas RS is largely affected by the degree of PR expression and is more sensitive than SUV in these specific tumors.

### Logistic regression analysis

Variables with p < 0.05 on univariate analysis—including SUV, tumor size, stage, PR expression, and Ki67—were entered as input variables in multivariate analysis in order to distinguish higher RS (≥26). Multivariate analysis revealed that SUV and PR expression remained independent variables associated with higher RS (Table 3). Among these, SUV demonstrated the highest odds ratio (OR = 100.62; 95% CI = 10.01–1003.64) for predicting higher RS on the multivariate analysis. The area under the receiver operating characteristic curve for continuous SUV was 0.928 (95% CI, 0.884–0.972, P<0.001) for distinguishing higher RS from lower RS (S2 Fig).

**Table 3:** Binary logistic regression analysis for factors associated with higher recurrence score (≥26).

Variables	Univariate (*P*)	Multivariate (*P*)	Odds Ratio	95% CI
**SUV**	<0.001	<0.001	100.62	10.01–1003.64
<4 vs. ≥4				
**Tumor size**	0.010	0.764	2.51	0.01–1026.26
≤2cm vs. >2cm				
**Nodal status**	0.251			
Negative or Micrometastasis vs. Positive				
**Stage**	0.034	0.785	2.29	0.01–885.23
I vs. II				
**Histologic grade**	<0.001	0.811	1.20	0.27–5.24
I or II vs. III				
**Estrogen receptor** ^**a**^	0.548			
Higher vs. Lower				
**Progesterone receptor** ^**a**^	<0.001	0.005	7.68	1.88–31.39
Higher vs. Lower				
**Ki67**	<0.001	0.083	3.15	0.86–11.56
<20% vs. ≥20%				

^a^ Higher, Allred score 5–8; Lower, Allred score 0–4.

### Adjuvant treatment according to RS or SUV

Of the 115 patients with low SUV tumors, 91.3% (95% CI, 86.2–96.5; *n* = 105) received endocrine treatment, and 8.7% (95% CI, 3.5–13.8; *n* = 10) received chemo-endocrine treatment (S2 Table). Of the 143 patients with lower RS tumors, 131 [91.6% (95% CI, 87.1–96.2)] received endocrine treatment alone, and 12 [8.4% (95% CI, 3.8–12.9)] received adjuvant chemotherapy followed by endocrine therapy.

Among patients with high risk factors, 24 of 51 patients (47.1%, 95% CI, 33.3–60.8) with high SUV received chemo-endocrine treatment, whereas 22 of 23 women (95.7%, 95% CI 87.3–100.0) with higher RS received chemotherapy. One patient was excluded from the analysis because of a refusal of adjuvant chemotherapy, despite a high RS of 38.

## Discussion

In our evaluation of biologic parameters with continuous function, we observed a certain degree of correlation between the SUV on 18F-FDG-PET-CT and RS in ER-positive, HER2-negative breast cancer. We showed that tumors with low SUV have a very high probability in having lower RS. In addition, our multivariate analysis revealed that high SUV (≥4) is independent factor associated with higher RS (≥26).

It is known that proliferation modules within multigene assays, including RS, are a common driving force behind the overall prognostic assay performance [14,15]. Also, previous studies provided evidence that tumors with increased glucoe uptkae have high proflierative propensities [9,10]. Therefore, some degree of concordance was expected between the SUV and RS. In our study, SUV also correlated with the Ki67 index and histologic grade, further confirming SUV provides a good reflection of tumor proliferation (S1 Fig). These findings provide biological evidence to support a prognostic value of SUV for luminal cancer, as suggested by our previous work.

Interestingly, we found an inverse correlation between the PR score and SUV. Recently, in a study of patients with ER-positive/HER2-negative disease, similar to those in our study, Prat et al. reported that semi-quantitative PR IHC analysis has improved in terms of identifying women with a good prognosis [16]. Currently, the use of a tumor’s PR status to identify luminal B/HER2-negative disease is recommended by St. Gallen’s guideline [4]. The absence of PR has long been regarded as an indicator of an impaired ER signaling pathway. In addition, the loss of PR is associated with the activation of cross-talk between the ER and growth factor signaling pathway [17]; this cross-talk may upregulate growth factor signaling and potentially contribute to increased glycolysis in PR-lacking tumors, as growth factor signaling plays a crucial role in the Warburg effect [18]. Further studies to elucidate the relationship between the loss of PR and increased glycolytic activity in this subset of breast cancer are warranted.

In analyses based on categorized RS and SUV, tumors with a low SUV tended to have a lower RS. In the low-SUV group, a high concordance rate (99.1%) was observed between a low SUV and lower RS. However, in patients with high SUV-tumors, a substantial discrepancy between the two markers was noted.

In further analyses of the discordance between two markers, the high SUV-lower RS group exhibited distinctive biological characteristics, compared with the concordant groups. The discordant group had either higher grade or higher Ki67 index when compared with the low SUV-lower RS group, and can therefore be considered a middle group between the two concordant groups in terms of pathologic factors such as the grade or Ki67 index. In accordance with these findings, the mean SUV increased stepwise among the three groups (data not shown).

By contrast, the average RS did not exhibit a stepwise pattern among the three groups. The mean RS in the discordant group was similar to that in the low SUV-lower RS group, but significantly lower than that in the high SUV-higher RS group (data not shown). Correspondingly, we observed similar PR scores between the low SUV-lower RS and the discordant group, whereas this score was significantly lower in the high SUV-higher RS group, compared with the discordant group (Fig 4). These results suggest that the RS might be very sensitive to PR expression, whereas the SUV might rely more strongly on proliferation indices such as Ki67 or the histologic grade. Indeed, in the tumors from our study, the absolute Pearson’s R value was higher for the correlation of the RS and PR score than for that of the RS and SUV (-0.735 vs. 0.555).

The strong interaction between PR expression and RS observed in our study can be explained by the fact that all of our patients had ER-positive, HER2-negative tumors. Because the RS was originally developed for ER-positive cancer cohorts, regardless of HER2 expression, and is strongly affected by the HER2 score, the influence of PR expression becomes more pronounced in our ER-positive, HER2-negative patients.

Taken together, our findings show that SUV, when used as a biologic parameter with continuous function, is associated with the RS in ER-positive, HER2-negative breast cancer. Despite the good accordance observed between SUV and RS, a certain group of patients exhibited discordance between these two markers at an individual level. These observations are predominantly attributed to biologic differences in these markers; specifically, RS is more sensitized to PR expression, whereas SUV is more strongly affected by histologic grade or Ki67 expression in ER-positive/HER2-negative cancers.

Furthermore, we investigated the actual use of adjuvant treatment according to the dichotomized SUV in a whole study population. We found that 105 of 115 (91.3%) in the patients with low SUV tumors had received endocrine treatment alone. Among patients with high SUV tumors, however, 47.1% received chemo-endocrine treatment, suggesting that according to current evidence, high SUV is not a determinant with regard to the addition of chemotherapy for patients with ER-positive/HER2-negative disease. Nevertheless, in addition to the high level of agreement between a low SUV and lower RS, the higher rate of endocrine treatment-only patients in the low-SUV group, which is comparable with lower RS (≤25), provides evidence of the potential clinical usefulness of SUV, at least with regard to identifying patients with a low recurrence risk.

One major limitation of our study is the absence of survival analyses among the divided groups by two markers because of the short follow-up duration. The clinical outcomes of our study population might help to refine prognostic discrimination according to these markers. Another limitation is selection bias; the RS is financially expensive, and therefore, RS were only obtained for a fraction of ER-positive/HER2-negative patients during the study period. In addition, we were unable to conduct a comparative analysis between the SUV and each of the 16 genes comprising the RS because we did not receive information regarding the expression levels of these genes. We note that information about the expression levels of other genes that comprise the RS could enhance our knowledge of the relationship between RS and SUV and our understanding of the biological characteristics underlying accordance or discordance between these factors.

Despite these limitations, we have provided novel evidence to support that the biologic parameter of glucose uptake magnitude correlates with RS; in turn, these findings support the prognostic value of the SUV for ER-positive, HER2-negative breast cancer. Further studies are warranted to determine the potential of SUV for the identification of risk groups among the patients with a lower RS.

## Conclusions

In conclusion, SUV as biologic parameters with continuous function was found to associate with RS in ER-positive, HER2-negative breast cancer. Further studies may reveal the biology underlying the discordance between the markers.

## Supporting information

S1 Table The baseline patient characteristics (DOCX)

S2 Table Adjuvant treatment according to RS or SUV (n = 161). (DOCX)

S1 Fig Relationship between the standardized uptake value (SUV) and continuous Ki67 or histologic grade. (A) SUV and continuous Ki67 (Pearson’s R = 0.371; *P* < 0.001) (B) SUV and histologic grade.
(TIF)

S2 Fig The area under the receiver operating characteristic curve for continuous SUV was 0.928 (95% CI, 0.884–0.972, P<0.001) for distinguishing higher RS from lower RS (S2 Fig). (TIF)

S1 File Raw Data. (XLS)
