# Supplementary material for: Comparison of standardized uptake value of 18F-FDG-PET-CT with 21-gene recurrence score in estrogen receptor-positive, HER2-negative breast cancer
Source: PLoS One. 2017 Apr 18;12(4):e0175048. doi: 10.1371/journal.pone.0175048 (PMC5395149; doi:10.1371/journal.pone.0175048)
Supplement: S1 Table — (DOCX) [file pone.0175048.s001.docx]

**S1 Table. Baseline characteristics**

|  | Number (%) |
| --- | --- |
| **Age** |  |
| Median (Range) | 48 (28-72) |
| **Histology** |  |
| IDC | 132 (81.5) |
| ILC | 15 (9.3) |
| Others ^a^ | 15 (9.3) |
| **Tumor size** |  |
| ≤2cm | 127 (78.4) |
| >2cm | 35 (21.6) |
| **Nodal status** |  |
| Negative | 141 (87.0) |
| Micrometastasis | 5 (3.1) |
| Positive | 16 (9.9) |
| **Stage** |  |
| IA | 111 (68.5) |
| IB | 5 (3.1) |
| IIA | 41 (25.3) |
| IIB | 5 (3.1) |
| **Histologic grade** |  |
| I | 36 (22.2) |
| II | 103 (63.6) |
| III | 21 (13.0) |
| Unknown | 2 (1.2) |
| **Estrogen receptor** |  |
| Positive (Allred score ≥2) | 162 (100.0) |
| **Progesterone receptor** |  |
| Positive (Allred score ≥2) | 138 (85.2) |
| Negative (Allred score <2) | 24 (14.8) |
| **HER2** |  |
| Negative | 112 (100.0) |
| **Ki67** |  |
| <20% | 133 (82.1) |
| ≥33 | 27 (16.7) |
| Unknown | 2 (1.2) |
| **Recurrence score** |  |
| Low (<18) | 92 (56.8) |
| Intermediate (18-30) | 57 (35.2) |
| High (31ia | 13 (8.0) |
| **SUV** |  |
| Low (<4) | 110 (67.9) |
| High (≥4) | 52 (32.1) |
| Median (Range) | 2.89 (0.93-14.19) |

Others include mucinous carcinoma, invasive cribriform carcinoma, invasive papillary carcinoma, and invasive ductal carcinoma with medullary feature.

Abbreviations: IDC, invasive ductal carcinoma; ILC, invasive lobular carcinoma; SUV, standardized uptake value
