# Supplementary material for: Comparison of standardized uptake value of 18F-FDG-PET-CT with 21-gene recurrence score in estrogen receptor-positive, HER2-negative breast cancer
Source: PLoS One. 2017 Apr 18;12(4):e0175048. doi: 10.1371/journal.pone.0175048 (PMC5395149; doi:10.1371/journal.pone.0175048)
Supplement: S2 Table — (DOCX) [file pone.0175048.s002.docx]

**S2 Table . Adjuvant treatment according to RS or SUV* (*n* = 161)**

|  | **Low SUV (*N*=110)** | **High SUV (*N*=51)** | ***P*-value** |  | **d-RS eUV**  **(*N*=138)** | **d-RS>25 ( *N*=23)** | ***P*-value** |
| --- | --- | --- | --- | --- | --- | --- | --- |
|  |  |  | **<0.001** |  |  |  | **<0.001** |
| **Endocrine alone (n=87)** | **100 (90.9)** | **27 (52.9)** |  |  | **126 (91.3)** | **1 (4.3)** |  |
| **Chemo-endocrine (n=24)** | **10 (9.1)** | **24 (47.1)** |  |  | **12 (8.7)** | **22 (95.7)** |  |

* One patient was excluded from the analysis because of refusal of adjuvant chemotherapy, despite a high recurrence score (RS = 38).

Abbreviations: SUV, standardized uptake value; d-RS, dichotomized recurrence score
