# Supplementary figures and images for: Comparison of standardized uptake value of 18F-FDG-PET-CT with 21-gene recurrence score in estrogen receptor-positive, HER2-negative breast cancer
Source: PLoS One. 2017 Apr 18;12(4):e0175048. doi: 10.1371/journal.pone.0175048 (PMC5395149; doi:10.1371/journal.pone.0175048)

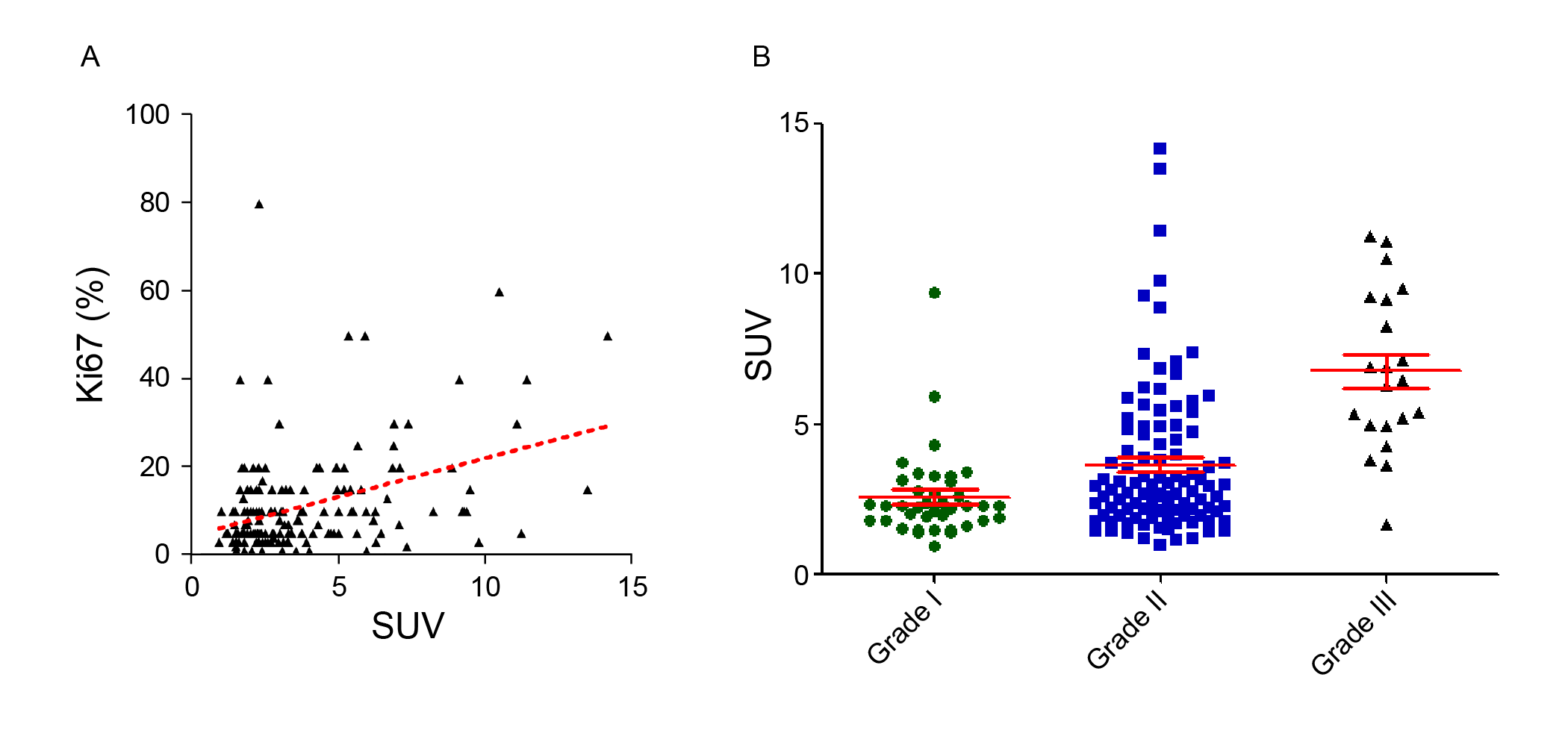

Supplement: S1 Fig — (A) SUV and continuous Ki67 (Pearson’s R = 0.371; P < 0.001) (B) SUV and histologic grade. (TIF) [file pone.0175048.s003.tif]

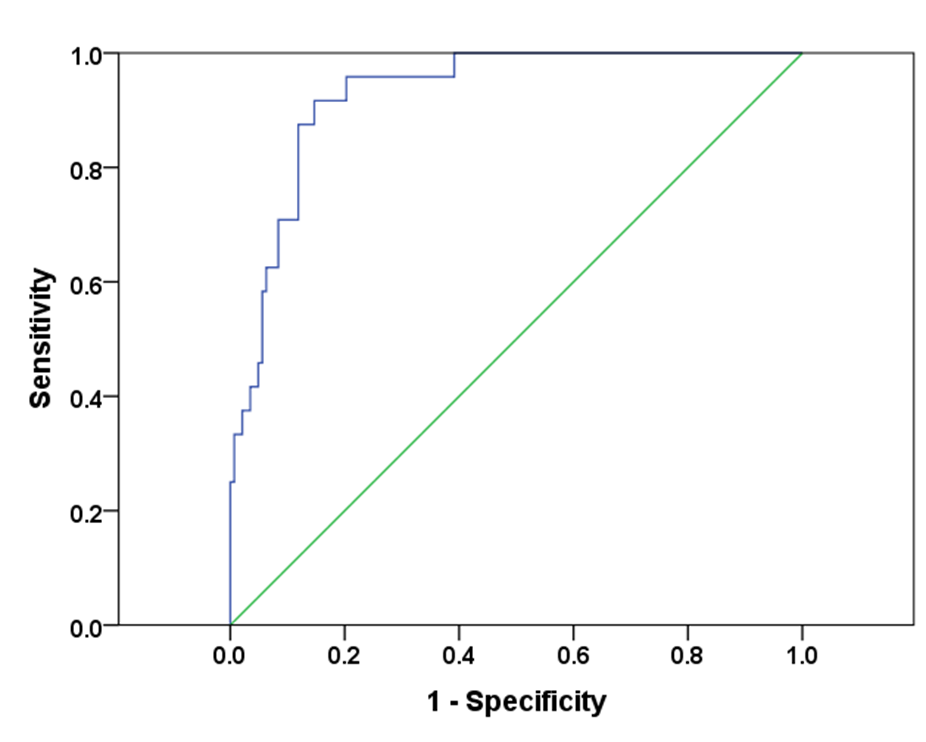

Supplement: S2 Fig — (TIF) [file pone.0175048.s004.tif]
